# The Secreted Ribonuclease SRE1 Contributes to *Setosphaeria turcica* Virulence and Activates Plant Immunity

**DOI:** 10.3389/fmicb.2022.941991

**Published:** 2022-07-08

**Authors:** Shidao He, Yufei Huang, Yanqiu Sun, Bo Liu, Suna Wang, Yuanhu Xuan, Zenggui Gao

**Affiliations:** ^1^College of Plant Protection, Shenyang Agricultural University, Shenyang, China; ^2^College of Life Sciences, Yan'an University, Yan'an, China; ^3^College of Landscape and Ecological Engineering, Hebei University of Engineering, Handan, China

**Keywords:** ribonuclease, *Setosphaeria turcica*, maize, cell death, plant immunity

## Abstract

During the plant infection process, pathogens can secrete several effectors. Some of the effectors are well-known for their roles in regulating plant immunity and promoting successful pathogen colonization. However, there are few studies on the ribonuclease (RNase) effectors secreted by fungi. In the present study, we discovered a secretable RNase (SRE1) in the secretome of *Setosphaeria turcica* that was significantly upregulated during the early stages of *S. turcica* infection in maize. Knockdown of *SRE1* significantly reduced the virulence of *S. turcica*. SRE1 can induce cell death in maize and *Nicotiana benthamiana*. However, unlike the conventional hypersensitive response (HR) caused by other effectors, SRE1 is not dependent on its signal peptide (SP) or plant receptor kinases (such as BAK1 and SOBIR1). SRE1-induced cell death depends upon its enzymatic activity and the N-terminal β-hairpin structure. SRE1 relies on its N-terminal β-hairpin structure to enter cells, and then degrades plant's RNA through its catalytic activity causing cytotoxic effects. Additionally, SRE1 enhances *N. benthamiana'*s resistance to pathogenic fungi and oomycetes. In summary, SRE1 promotes the virulence of *S. turcica*, inducing plant cell death and activating plant immune responses.

## Introduction

Northern corn leaf blight (NCLB) is caused by *Setosphaeria turcica* and is one of the major foliar diseases in maize production areas worldwide (Jackson-Ziems, 2016; Meng et al., 2022). Yield losses usually range from 15 to 30%, up to 50% in severe cases (Raymundo and Hooker, 1981; Perkins, 1987). Between 2012 and 2015, NCLB is estimated to have caused a total loss of 27.9 million tons of maize in the United States and Ontario, Canada (Mueller et al., 2016). The most severe infections of this disease occur before harvest and spread during the filling period (Carson, 1995; Kotze et al., 2019). The optimal conditions for the onset of the disease include temperatures between 17 and 27°C, a dew period of at least 4 h, and a relative humidity (RH) of at least 90% or more (Levy, 1983; Bentolila et al., 1991; White, 1999).

Some qualitative resistance (R) genes have been previously identified in maize, including *Ht1, Ht2, Ht3, HtN, Htm1, Htn1, HtP, ht4*, and *rt* (Welz and Geiger, 2010; Hurni et al., 2015). The ability to overcome these R-genes can be used to classify the physiological subspecies of *S. turcica*. Isolates of race 23N, for instance, can overcome resistance conferred by the *Ht2, Ht3*, and *HtN* R-genes in maize plants. We discovered 16 physiological subspecies in 883 monoconidial isolates of *S. turcica* from Northeast China between 2007 and 2017 (Ma et al., 2021). Due to changes in pathogen populations, qualitative resistance frequently leads to disease resistance loss. As a result, quantitative resistance breeding has emerged as the key strategy for combating NCLB (Hurni et al., 2015).

Histopathological studies have been reported in *S. turcica*-infected maize (Kotze et al., 2019). After germination, the conidia of the *S. turcica* track developed appressorium and then produced penetration pegs to enter the maize epidermis directly (Knox-Davies, 1974). The fresh hyphae then grow in the epidermal cells or intercellular spaces and slowly expand to adjacent cells, while some hyphae grow and colonize the xylem vessels, producing only mild chlorotic spots on the leaves (Hilu and Hooker, 1963). When the mycelium in the xylem vessels interacts with the susceptible host, it proliferates more quickly, spreading to nearby mesophyll cells, culminating in the development of typical fusiform necrotic lesions (Kotze et al., 2019).

The molecular mechanisms of *S. turcica* pathogenicity mainly involve the cell signal transduction pathway, melanin biosynthesis, and stress response to environmental stress. For example, StSte12 (a downstream protein of the Fus3/Kss1-homolog of the mitogen-activated protein kinases cascade) and StRas2 (a type of monomeric GTPase) have been implicated in appressorium development (Zhang et al., 2012; Gu et al., 2014). In *S. turcica*, StPKA-c (the catalytic subunit of cAMP-dependent protein kinase A) and Stgg1 (a G protein) are important for conidiation (Shen et al., 2013; Li et al., 2018). Melanin plays a role in the pathogenic process of *S. turcica* as well. STMR1, a zinc finger transcription factor that regulates the expression of dihydroxy naphthalene (DHN) melanin synthesis pathway genes, has been found to alter pathogenicity and melanin synthesis in *S. turcica*. The *STMR1* gene mutant showed a significant loss of pathogenicity (Zhang et al., 2022). Additionally, a recent study has shown that to maintain genomic integrity and cell survival, *S. turcica* responds to genotoxic load by activating the S-phase checkpoint through *StATR* (Zeng et al., 2020).

Plants have a two-layer immune system. Pattern-triggered immunity (PTI) is the first layer immune system, which is activated by pattern recognition receptors (PRRs) on the cell membrane that recognizes pathogen-associated molecular patterns (PAMPs). The second layer immune system is effector-triggered immunity (ETI), which is activated directly or indirectly via nucleotide-binding of leucine-rich repeat receptors (NLRs) located in the cell that senses the pathogens (Cui et al., 2015; Couto and Zipfel, 2016; Ngou et al., 2022). Recent research has shown that these two immune responses are interconnected and coordinated (Ngou et al., 2021; Pruitt et al., 2021; Yuan et al., 2021; Zhai et al., 2022). Phosphorylation of the NADPH oxidase RBOHD was discovered to enhance the formation of reactive oxygen species (ROS) and to be an early key signaling event linking PRR and NLR-mediated immune systems, whereas BIK1 phosphorylation was required for full RBOHD activation, gene expression, and bacterial resistance in the ETI system. Furthermore, NLR signaling accelerated the transcription and protein production of PTI signaling factors (Yuan et al., 2021). Studies have shown that when plant cell surface receptors recognize pathogens, they turn on several protein kinases and NADPH oxidases. Intracellular receptors boost the activation of these proteins by making more proteins available through several mechanisms. In addition, activation of cell surface receptors greatly enhances intracellular receptor-dependent hypersensitivity. As a result, plants' cell surface and intracellular receptor-activated immune pathways reinforce each other, resulting in enhanced defense against pathogens (Ngou et al., 2021). Another study found that the EDS1-PAD4-ADR1 module can play a role in membrane and intracellular LRR receptor-activated defense signaling cascades and regulate plant immunity through PTI and ETI (Pruitt et al., 2021). According to a recent study, the deubiquitinase PICI1 functions as an immunological hub for PTI and ETI in rice. PICI1 deubiquitinates and stabilizes methionine synthetase, which largely activates methionine-mediated immunity via the biosynthesis of the plant hormone ethylene. PICI1 is targeted for degradation by *Magnaporthe grisea* effectors, including AvrPi9, to inhibit PTI. NLRs in the plant immune system, such as PigmR, protect PICI1 from effector-mediated degradation to restart the methionine-ethylene cascade (Zhai et al., 2022). These investigations indicated that the two key immunity processes in plants, PTI and ETI, do not work separately, but rather have a synergistic impact of mutual amplification, ensuring that plants can produce a long-lasting and powerful immune response in response to pathogen invasion.

The functions of many effector proteins in plant pathogens have been previously reported, such as AvrPiz-t, Cmu1, Slp1, and XEG1 (Li et al., 2009; Djamei et al., 2011; Mentlak et al., 2012; Ma et al., 2015). The sequencing of the *S. turcica* genome has enabled the study of predicted functions of candidate effector proteins (Condon et al., 2013; Cao et al., 2020). *S. turcica's* first successful protein R-gene model was recently described. AVRHt1 (protein ID 179218) was anticipated to be a hybrid polyketide synthase (PKS) non-ribosomal synthetase (NRPS) identified by maize R-gene *Ht1* (Mideros et al., 2018). A transcriptome study revealed different expression patterns of different genes in the biotrophic and necrotrophic stages of *S. turcica* (a hemibiotrophic pathogen) and identified 346 candidate effector proteins, including the previously reported ones StEcp6 (Xue et al., 2013; Human et al., 2020). For example, StCFEM12 can inhibit programmed cell death (PCD) induced by INF1, with StACE1 acting as an oppressor-coupled effector protein that causes cell necrosis in *Nicotiana benthamiana* (Wang et al., 2021; Meng et al., 2022). However, little is known about the roles of the key effector protein in the *S. turcica* infection process.

RNase (secreted ribonuclease) is an enzyme that plays an important role in pathogen-host interactions in nature. Among them, two types of RNase have been widely concerned: ribosome-inactivating proteins (RIPs) and fungal ribotoxins (Lacadena et al., 2007; Walsh et al., 2013). These RNases can traverse the cell membrane and engage with the sarcin-ricin loop (SRL) on the large ribosomal subunit, preventing protein synthesis and causing cell death (Olombrada et al., 2017). Similarly, ribotoxins are produced by fungi when needed for survival and colonization. For example, α-sarcin, restrictocin, and Aspf1 are from three different *Aspergillus* species, and these proteins are highly conserved. Aspf1 belongs to fungal RNase T1 (mainly *Aspergillus* and *Penicillium*), which stands out for its cytotoxicity (Olson and Goerner, 1965; Makarov and Ilinskaya, 2003; Lacadena et al., 2007). Fg12 is an RNase from *Fusarium graminearum* that contributes to the pathogen's virulence and induces plant cell death light-dependently (Yang et al., 2021). Zt6 is the first RNase effector from *Zymoseptoria tritici* to be shown to have dual roles, possibly contributing to wheat cell death and maybe playing a role in antimicrobial competition and niche protection (Kettles et al., 2018). UV_1423 is predicted to be a fungus-specific RNase in *Ustilaginoidea virens*, which causes rapid and severe cell death in *N. benthamiana* depending on the active site of the enzyme (Fang et al., 2016). Furthermore, CSEP0064/BEC1054 is a pseudoenzyme that can compete with plant RIPs for ribosomal binding, thereby inhibiting cell death and maintaining pathogen growth (Pennington et al., 2019).

In this study, we conducted a secretome investigation of *S. turcica* and discovered 90 potential effector proteins. The gene encoding one of the secreted ribonucleases (SRE1) was substantially increased in the early stages of maize infection by *S. turcica*, drawing our attention. SRE1 is a member of the RNase F1 family, extensively conserved in plant pathogenic fungi and, depending on its enzymatic active site, can induce severe cell death in *N. benthamiana*. We found that the β-hairpin structure at the N-terminal of SRE1 was essential for its virulence. In addition, we demonstrated that SRE1 is essential as a virulence factor of *S. turcica* during maize infection and can also activate plant defense responses, thereby improving plant resistance to oomycetes and fungi.

## Materials and Methods

### Strains and Plant Growth Conditions

Wild type *S. turcica* isolate TL-5 was cultured in potato dextrose agar (PDA) medium and grown at 28°C in the dark. *Phytophthora capsica* and *Botrytis cinerea* strains were cultured in 10% V8 juice agar at 24°C in the dark. The *Escherichia coli* strain DH5α used to propagate the plasmid was grown in a liquid Luria Bertani (LB) medium at 37°C, while *Agrobacterium tumefaciens* strain GV3101 in LB medium at 28°C was used to infiltrate plants. Maize and *N. benthamiana* were grown and maintained in a greenhouse at 26°C with a 16 h light/8 h dark cycle.

### Agroinfiltration Assays

*A. tumefaciens* GV3101 strain was cultured and collected by centrifugation at 7,500 g, then resuspended in the injection buffer (200 μM acetosyringone, 10 mM MES pH 5.7, 10 mM MgCl_2_) and adjusted to OD_600_ = 0.6. For co-expression, the two cultures carrying the suitable constructs were mixed in a 1: 1 ratio to OD_600_ = 0.6 for each. After incubation in the dark at 28°C for more than 2 h, the culture was progressively infiltrated from the back of four-week-old *N. benthamiana* leaves with a 1 ml syringe with the needle detached.

### ROS and Callose Staining

ROS accumulation in plants was visualized using diaminobenzidine staining. Agro-infiltrated *N. benthamiana* leaves were soaked in a 1 mg/mL solution of diaminobenzidine (adjusted to pH 3.8 with hydrochloric acid) and incubated in the light at room temperature for 8 h. The samples were submerged in 100% ethanol and maintained in a water bath (100°C) until all the chlorophyll was extracted. Finally, the samples were immersed in 20% glycerol for microscopic observation. Callose deposition in plants was visualized using aniline blue staining. Agro-infiltrated *N. benthamiana* leaves were fixed and de-stained overnight in 1: 3 acetic acid/ethanol, washed with 150 mM K_2_HPO_4_ for 30 min, and then incubated in 150 mM K_2_HPO_4_ and 0.01% aniline blue for at least 2 h in the dark and imaged under fluorescence microscopy.

### Prokaryotic Expression and Purification of Recombinant Proteins

The sequence of *SRE1* and *SRE*1^67A^ without the signal peptide were connected to the prokaryotic expression vector pGS21T and transformed into *E. coli* BL21 (DE3) strain. A single clone was selected from the transformation plate and inoculated into liquid LB media (including ampicillin 50 mg/mL), which was cultured at 37°C for about 4 h until the OD_600_ was around 0.6, and the expression was stimulated by adding IPTG at a final concentration of 0.5 mM. After expansion, cells were collected in 1.6 L liquid medium and resuspended in buffer (20 mM Tris-HCl pH 8.0, 50 mM NaCl, 0.1% Triton-100), lysed by sonication (600–800 W, 30 min), centrifuged (10,000 rpm, 15 min). Recombinant proteins were loaded in batches of 5 or 10 mL into GSTPrep FF 16/10 columns (Clontech, Japan) with Glutathione Sepharose 4 Fast Flow and purified in combination with the ÄKTA go (Cytiva, United States) protein purification system at a constant flow rate of 0.5 mL/min. The samples were then rinsed to baseline with sufficient equilibration buffer (50 mM Tris-HCl, pH 8.0), eluted with the elution buffer (10 mM Glutathione, 50 mM Tris-HCl, pH 8.0), and the flow-through was then collected. Finally, the eluted protein was dialyzed against 20 mM Tris pH 8.0 and 50 mM NaCl. SRE1 and SRE1^67A^ protein concentrations were determined using the BCA protein concentration assay kit after dialysis and were found to be 0.4 and 0.8 mg/mL, respectively.

### RNA Extraction and the qPCR Analysis of Gene Expression

The total RNA of *N. benthamiana* or maize leaves was extracted using the MiniBEST Universal RNA Extraction kit (Takara, Japan). PrimeScript^TM^ RT reagent kit (Takara, Japan) was used to synthesize cDNA from total RNA samples. The qPCR total reaction volume was 25 μl, including 2 μl specific primers (Supplementary Table 2), 2 μl cDNA or gDNA template, 6.5 μl sterilized water, 12.5 μl TB Green Premix Ex Taq II (Takara, Japan). Three reactions were prepared for each sample-primer pair combination. All reactions were run on the CFX96 system using the following thermal cycle: 95°C pre-denaturation for 30 s, then 95°C for 10 s, extension at 60°C for 30 s, 40 cycles. Specific internal reference primers were used to normalize the transcription level between samples (Supplementary Table 2). Three biological replicates were included for each treatment. Relative expression values were calculated using the 2^−ΔΔCt^ method (Schmittgen, 2008).

### Plasmid Construction

PCR amplified all fragments with the Phusion High-Fidelity DNA Polymerase (Thermofisher, United States) from the cDNA samples. These fragments were constructed into the pGR107 using the ClonExpress II One Step Cloning Kit (Vazyme, China) for transient expression. For VIGS analysis, the fragment was cloned into pTRV-RNA2 by double digestion and ligation. The primers used in this study are listed in Supplementary Table 2.

### Yeast Signal Sequence Trap

Previous investigations were used to develop the yeast signal-peptide detection system (Jacobs et al., 1997). The sequence of the signal peptide of SRE1 was cloned into an invertase vector pSUC2 coding for the deletion of the secretory signal peptide, and the vector was then transformed into the invertase-deficient yeast YTK12 strain. The CMD-W medium screened positive yeast transformants (0.67% yeast N base without amino acids, 0.075% W dropout supplement, 2% sucrose, 0.1% glucose, 2% agar). The yeast transformants that recovered secretory function were evaluated using YPRAA media (1% yeast extract, 2% peptone, 2% raffinose, 2 mg/mL antimycin A). The empty vector pSUC2 and the vector Avr1b-SP served as negative and positive controls, respectively.

### VIGS in *N. benthamiana*

The vectors pTRV2-NbBAK1, pTRV2-NbSOBIR1, pTRV2-GFP, and pTRV2-PDS, were constructed and transformed by heat shock into the *A. tumefaciens* GV3101 strain. *Agrobacterium* cultures containing pTRV-RNA1 and pTRV-RNA2-gene constructs were mixed in a 1: 1 ratio and infiltrated into true leaves of five-leaf stage *N. benthamiana* seedlings at a concentration of OD_600_ = 0.6. After 3 weeks, the top two largest true leaves were used for transient expression and silencing efficiency assay. The silencing efficacy of VIGS was visualized using pTRV2-PDS, with pTRV2-GFP serving as a control. qPCR was used to determine the effectiveness of gene silencing (Liu et al., 2021).

### Infection Assays

The conidia of *S. turcica* wild-type strain TL-5 were collected after 20 days of culture on PDA media in the dark. The conidia suspension culture (5.0 × 10^4^/mL) was inoculated on corn leaves at the six-leaf stage, placed in an incubator at 20°C and 80% RH for 5 days, the diameter of the lesions was measured on the 5 days after inoculation, and the relative biomass of *S. turcica* was determined using qPCR. The fresh mycelia of *P. capsica* and *B. cinerea* were inoculated on the back of *N. benthamiana* leaves, infiltrated with *Agrobacterium* 24 h before inoculation to express the corresponding protein, and then the plants were placed in an incubator at 24°C and 80% RH. Lesion diameters were measured 48 h after inoculation. Genomic DNA was extracted from diseased tissue of equal size, and relative pathogen biomass was measured using qPCR.

### Protein Extraction and Western Blotting

*Agrobacterium* transient expression was performed using leaves of four-week-old *N. benthamiana* plants. Leaves were collected 48 h after infiltration and ground in liquid nitrogen. Lysis buffer (50 mM Tris pH 7.4, 150 mM NaCl, 1% Triton X-100, 1% sodium deoxycholate, 10% glycerol and 0.1% SDS) containing protease inhibitor cocktail was used for protein extraction. The mixture was centrifuged at 13,000 rpm for 15 min at 4°C and then at 100°C for 5 min. Subsequently, the specimens were separated using sodium dodecyl sulfate-polyacrylamide gel electrophoresis (SDS-PAGE) and analyzed through a Western blot assay.

### Bioinformatics Analysis

The protein domains were searched by HMMER3 (https://myhits.sib.swiss/cgi-bin/hmmer3_search), and the proteins identified by mass spectrometry were annotated by the Uniprot online database (https://beta.uniprot.org). Signal peptide prediction was performed using the online SignalP-5.0 server (https://services.healthtech.dtu.dk/service.php?SignalP-5.0). Homologous proteins of SRE1 were acquired from the Uniprot online database and aligned through the CLUSTALW server (https://myhits.sib.swiss/cgi-bin/clustalw). Phylogenetic trees were constructed in MEGA software (version 10.0) with the maximum likelihood of using 1,000 bootstrap replicates to ascertain the reliability of a given branch pattern in the NJ tree.

### Accession Numbers

The gene sequences cloned in this study were uploaded to the GenBank database include *SRE1* (SETTUDRAFT_163271), *MgSRE1* (MGG_10720), *UmSRE1* (UMAG_10881), *VdSRE1* (VDAG_00107), *CgSRE1* (GLRG_09613), and *FoSRE1* (FOMG_09672). The homologous protein IDs of SRE1 presented in this study were obtained for other fungi, including: *Sclerotinia sclerotiorum* (SS1G_06394), *B. elliptica* (BELL_0189g00140), *Pyrenophora tritici-repentis* (PTRG_05531), *Phaeosphaeria nodorum* (JI435_102410), *Valsa mali* (VMCG_07534), *F. graminearum* (FG11190.1), *Z. tritici* (MYCGRDRAFT_38105), *Moniliophthora perniciosa* (MPER_12178), *Cochliobolus sativus* (COCSADRAFT_36381), *Sporisorium reilianum* (sr12878), *Penicillium digitatum* (PDIG_67970), *Trichoderma harzianum* (M431DRAFT_556268), and *Neurospora crassa* (nc3877848).

## Results

### Secretome Analysis of the *S. turcica*

**Figure 1:**
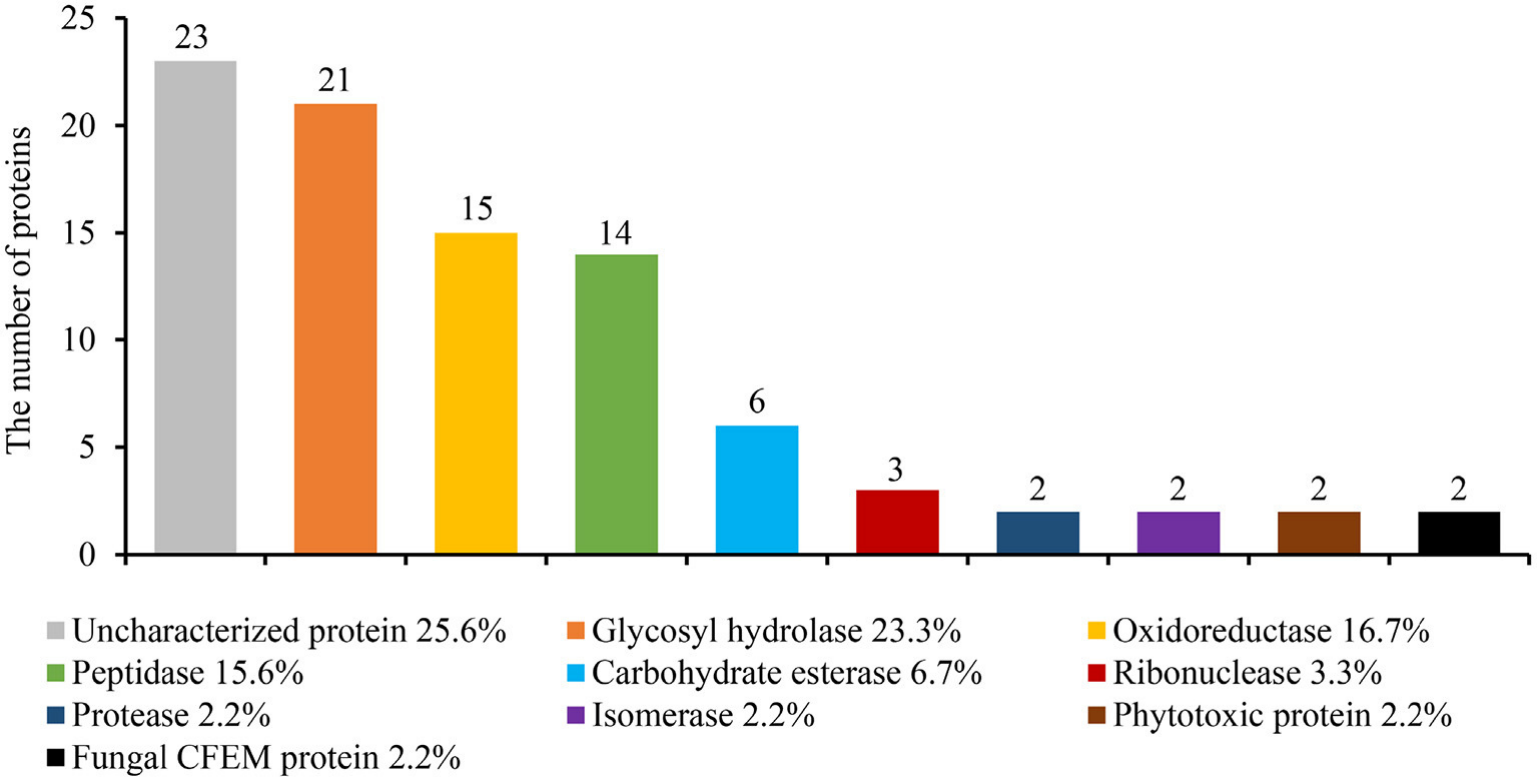
Identifying the *S. turcica* secreted proteins by liquid chromatography-mass spectrometry (LC-MS/MS). *S. turcica* was cultivated for 5 days in a potato liquid medium, the supernatant was collected, and the mycelium and impurities were removed through ultrafiltration tubes. The composition of *S. turcica* secretory proteome was analyzed by LC-MS/MS. The different colors in the column chart represent different protein families. The numbers and the percentages of different proteins are also shown.

The genome of *S. turcica* encodes over 200 small secreted proteins, which are also known as putative effectors (Condon et al., 2013). The fungus was pre-cultured in potato dextrose broth for 5 days to investigate if these putative effectors are secreted extracellularly. Centrifugal filters (3kDa) were used to extract the mycelia and impurities. The secretions were then analyzed using liquid chromatography-tandem mass spectrometry (LC-MS/MS). Finally, 90 proteins were identified in the *S. turcica* strain TL-5 secretome (Supplementary Table 1). The proteins were predicted by SignalP-5.0 (Yang et al., 2021), which revealed that they all contained a secretory signal peptide at the N-terminal. Most of the identified proteins belonged to enzymes with different functions, including glycosyl hydrolase (23.3%), oxidoreductase (16.7%), peptidase (15.6%), carbohydrate esterase (6.7%), RNase (3.3%), protease (2.2%), isomerase (2.2%). In addition, uncharacterized protein (25.6%) and several phytotoxic proteins (2.2%) and fungal CFEM proteins (2.2%) were included (Figure 1).

### *SRE1* Encodes a Secreted RNase That Is Highly Upregulated During the *S. turcica* Infection

We used RNA sequencing data from maize infected with *S. turcica* strain TL-5 to screen for effector encoding transcripts to study the probable function of the proteins secreted by *S. turcica* (unpublished content). The transcriptome data demonstrated that one of these effector-encoding genes, *SRE1*, was highly upregulated during infection, suggesting a potential role of SRE1 during infection.

**Figure 2:**
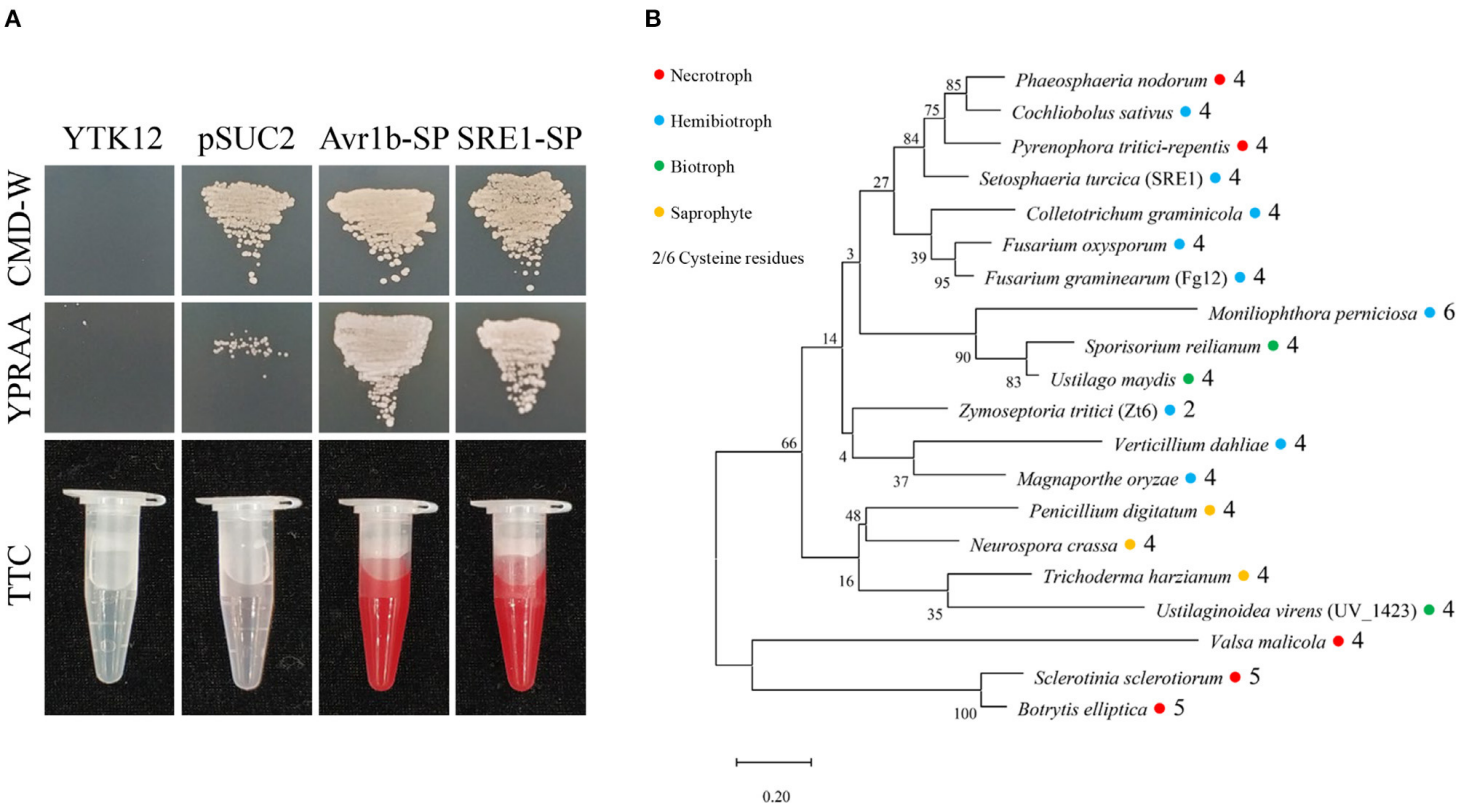
Secretory function and phylogenetic analysis of *S. turcica* effector SRE1. **(A)** The yeast-signal peptide trap system was used to verify the signal peptide function of SRE1. A CMD-W medium screened the yeast strain YTK12 carrying the pSUC2 vector. The signal peptide with secretion function can make up the secretion defect of YTK12 invertase so that YTK12 can grow in the YPRAA medium. The invertase activity was detected by reducing TTC to insoluble red TPF. The Avr1b-SP and the empty vector pSUC2 were used as positive and negative controls, respectively. **(B)** Phylogenetic tree of SRE1 homologs in fungi of different trophic types. The sequences were derived from the Uniprot database and aligned using CLUSTALW. The phylogenetic tree was constructed in MEGA. The colored circles represent different trophic types of fungi, and the numbers represent the number of cysteines.

*SRE1* encodes a fungal secretory RNase, which contains 135 amino acids and four cysteine residues, and the amino acid positions 1-17 were predicted as a secretory signal peptide (Supplementary Figure 1). To verify the secretory function of SRE1, we performed yeast signal trap assays (Jacobs et al., 1997). The sequence of SRE1's signal peptide was fused with the vector pSUC2 expressing invertase without its signal peptide. Afterwards, the recombinant plasmid was transformed into the translocase-deficient yeast strain YTK12 and cultured in disaccharide media. The SRE1-SP strain developed in the YPRAA medium in the same way as the positive control Avr1b-SP. The secretory activity of invertase was restored, thereby reducing 2,3,5-triphenyl tetrazolium chloride (TTC) to insoluble red 1,3,5-triphenyl formazan (TPF) (Figure 2A). Based on these findings, the signal peptide of SRE1 is sufficient for the secretion of invertase, indicating that SRE1 is a secreted protein in *S. turcica*.

Maize at the six-leaf stage was inoculated with *S. turcica*, and infected samples were taken at different infection stages to better understand the expression dynamics of *SRE1*. Quantitative real-time polymerase chain reaction (qRT-PCR) analysis showed that *SRE1* expression was strongly induced at the early stage of *S. turcica* infection in maize and peaked 48 h after infection (Supplementary Figure 3). These findings suggest that *SRE1* expression is highly upregulated early in infection, implying a link between *S. turcica* pathogenicity and *SRE1* expression.

### SRE1 and Its Homologs in Other Pathogens Can Induce Cell Death in *N. benthamiana*

The homologs of SRE1 proteins were found in a variety of trophic types of fungi, including a biocontrol fungus, *T. harzianum*, according to phylogenetic analyses. Cysteine levels in most fungi ranged from four to six, but only two in *Z. tritici*. These cysteines produced disulfide bonds that may affect protein folding (Figure 2B). Analysis of the sequences of SRE1 homologs revealed large variation in the N-terminus of the mature protein, while the RNase functional domain was relatively conserved (Supplementary Figure 2).

**Figure 3:**
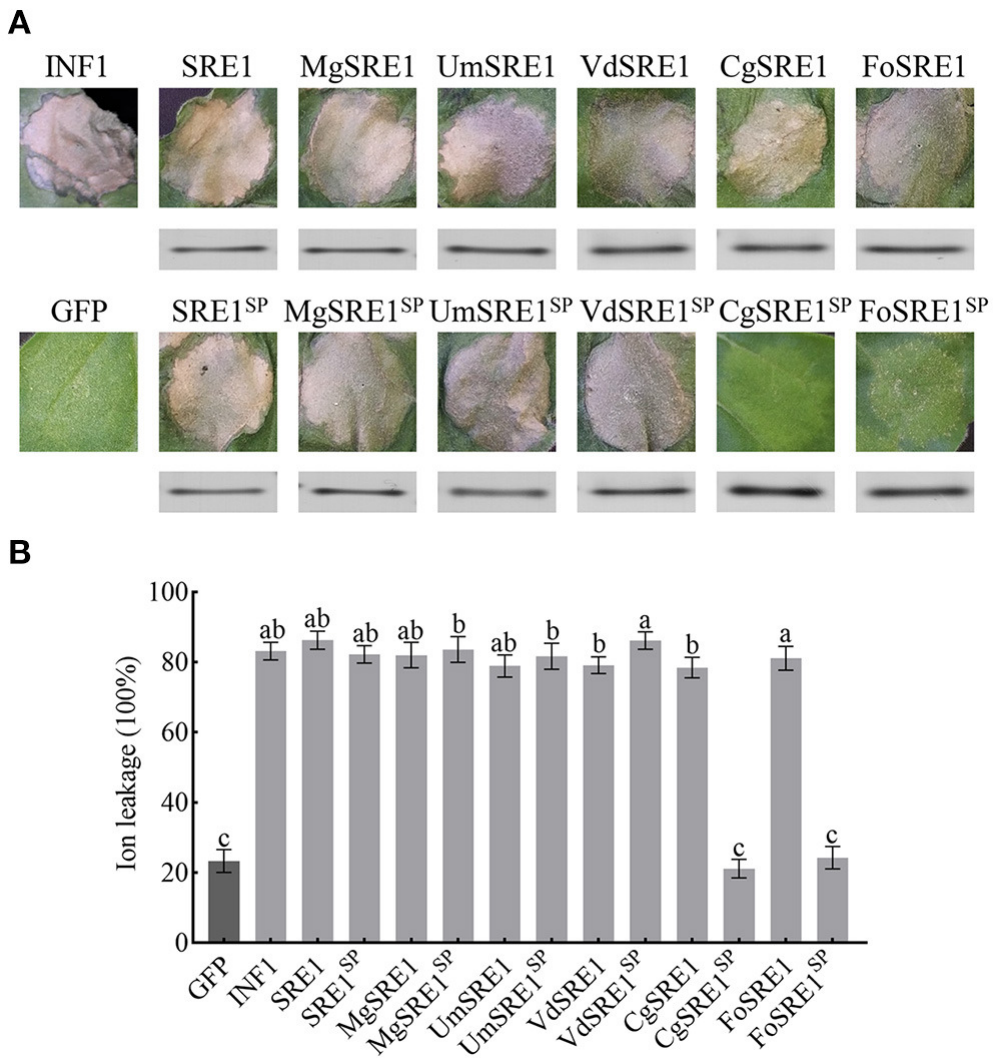
The homologous genes of SRE1 from different pathogenic fungi induces cell death in *N. benthamiana*. **(A)** Transient expression of SRE1, SRE1 homologous proteins, and signal peptide deletion mutants in *N*. *benthamiana*. INF1 and GFP were used as positive control and negative control, respectively. Western blot analysis of 3 × HA-tagged proteins expressed in *N*. *benthamiana* leaves. **(B)** Quantitative analysis of cell death by electrolyte leakage. The bars are the average of three independent experiments, and the error bars represent the standard deviations. Different letters at the top of the bars indicate significant differences (one-way ANOVA; *P* < 0.05).

Full-length genes were cloned into the pGR-107 vector, which contained a C-terminal 3 × HA tag, to verify the function of SRE1. Agroinfiltration was used to transiently express the *SRE1* gene in *N. benthamiana* leaves. The results showed that SRE1, like positive control INF1, induced severe necrosis in *N. benthamiana* 3 days after injection (Figure 3A). Meanwhile, *MgSRE1, UmSRE1, VdSRE1, CgSRE1*, and *FoSRE1* genes from *M. grisea, Ustilago maydis, Verticillium dahliae, Colletotrichum graminicola*, and *F. oxysporum*, respectively, were also cloned. These genes belong to the same RNase F1 family as *SRE1* of *S. turcica*. The results demonstrated that, like the *S. turcica* SRE1 and the positive control INF1, all of these homologs elicited substantial necrotic reactions in *N. benthamiana* 3 days after injection (Figure 3A).

To determine whether RNase-induced necrosis is dependent on signal peptides, the signal peptides of SRE1 and SRE1 homologs were eliminated and expressed in *N. benthamiana* to obtain the mutants SRE1^SP^, and MgSRE1^SP^, UmSRE1^SP^, VdSRE1^SP^, CgSRE1^SP^, and FoSRE1^SP^. Interestingly, MgSRE1^SP^, UmSRE1^SP^, and VdSRE1^SP^ induced cell death in *N. benthamiana*, but CgSRE1^SP^ and FoSRE1^SP^ failed to induce cell death (Figure 3A). Immunoblot analysis of the total protein extract from the agroinfiltrated leaf area confirmed the effective protein translation of SRE1, SRE1 homologs, and the mutants (Figure 3A). Electrolyte leakage was used to reflect the extent of the cell death (Figure 3B). In conclusion, RNases of various phytopathogenic fungi can cause cell death in *N. benthamiana*, depending on their signal peptides in some cases and not in others.

### SRE1 Induces Cell Death in *N. benthamiana* Independent of NbBAK1 and NbSOBIR1

**Figure 4:**
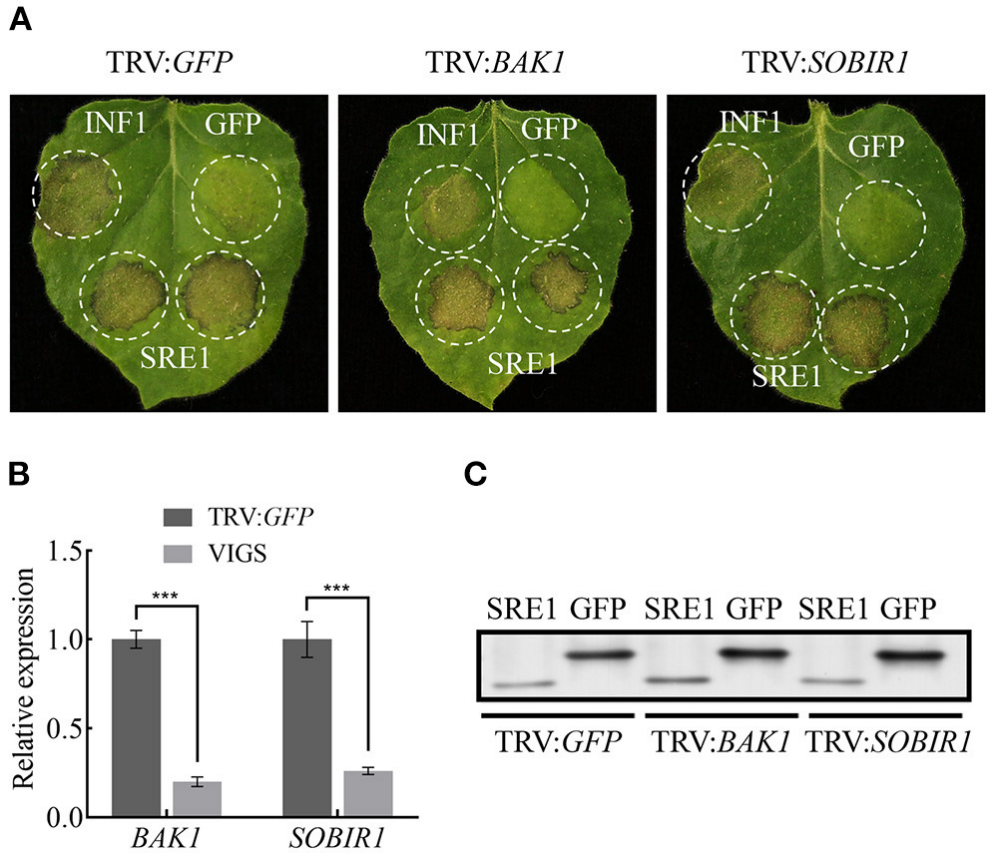
SRE1-induced cell death is BAK1/SOBIR1-independent. **(A)** The TRV-mediated gene silencing system was used to verify that SRE1-induced cell death is independent of BAK1/SOBIR1. INF1 and GFP were positive and negative controls, respectively. **(B)** Expression levels of *BAK1* and *SOBIR1* after silencing were detected by qPCR. The Student's *t*-test was carried out to determine the significance of the difference. Error bars indicate the standard deviations of three biological replicates. ***Indicates a significant difference at a *P*-value of < 0.001. **(C)** Western blot analysis of proteins expressed in silenced *N. benthamiana* leaves.

Many effector proteins induce cell death in *N. benthamiana* dependent on the Brassinosteroid Insensitive 1 (BRI1)-Associated Receptor Kinase 1 (BAK1) and Suppressor of BIR1-1 (SOBIR1) receptor-like kinases (RLKs) (Russinova et al., 2004; Zhang et al., 2013). *NbBAK1* and *NbSOBIR1* were silenced in *N. benthamiana* leave using the virus-induced gene silencing (VIGS) system, and qPCR was used to validate the silencing of *NbBAK1* and *NbSOBIR1* 3 weeks after VIGS-mediated gene silencing. These leaves were then infiltrated with SRE1, positive control INF1, and negative control GFP. Immunoblotting analysis confirmed that SRE1 was expressed in *N. benthamiana* leaves inoculated with pTRV2: *BAK1*, pTRV2: *SOBIR1*, and pTRV2: *GFP*, but the results indicated that SRE1 can still induce cell death in *NbBAK1*- or *NbSOBIR1*-silenced plants (Figures 4A–C). In summary, SRE1-induced cell death was independent of the BAK1 and SOBIR1 RLKs.

### The Enzymatic Activity of SRE1 Is Necessary for Its Cytotoxicity

**Figure 5:**
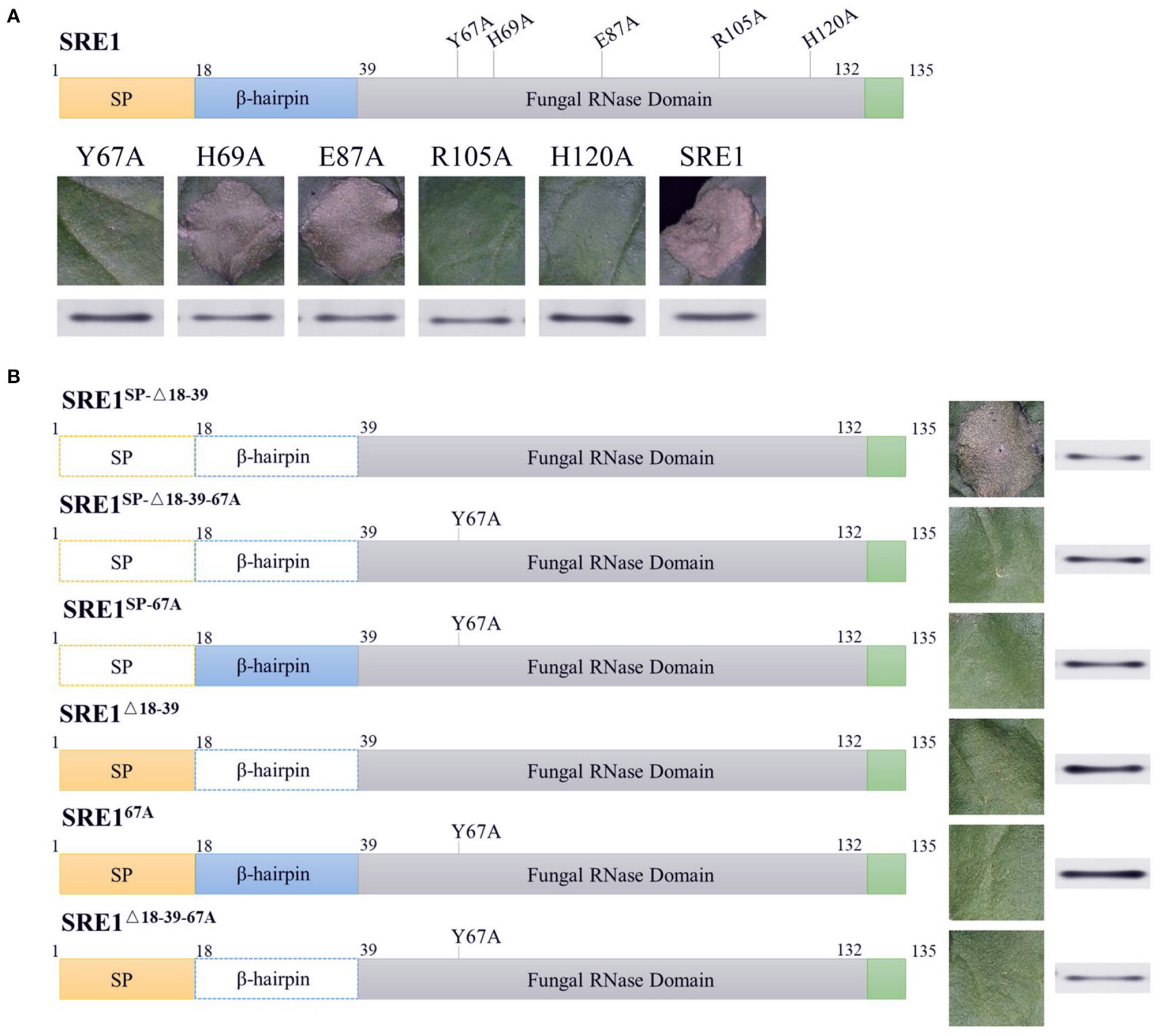
The cytotoxicity of SRE1 depends on its enzymatic active site and N-terminal β-hairpin structure. **(A)** *N. benthamiana* leaves were infiltrated with *A. tumefaciens* carrying the SER1 enzyme active site mutants. Western blot analysis of 3 × HA-tagged proteins expressed in *N. benthamiana* leaves. **(B)** *N. benthamiana* leaves were infiltrated with *A. tumefaciens* carrying different mutants of the SER1. The superscripts SP, Δ18-39, and 67A, represent SRE1 missing the signal peptide, N-terminal β-hairpin, and catalytic site Y67, respectively. Western blot analysis of 3 × HA-tagged proteins expressed in *N. benthamiana* leaves.

RNases cleave phosphodiester bonds in RNA and are essential for non-specific RNA degradation and numerous forms of RNA processing. This conserved protein domain family members belong to the superfamily of microbial RNases, predominantly guanyl-specific nucleases. The amino acid positions 35-132 of SRE1 were predicted to be fungal type RNase domain (Interpro access number: cd00606) by National Center for Biotechnology Information (NCBI) database, which contained five catalytic sites at positions 67, 69, 87, 105, and 120 (Supplementary Figure 1). The five amino acid residues (Y67, H69, E87, R105, and H120) were individually mutated to alanine residues to observe if SRE1 induces cell death based on its enzymatic activity (Figure 5A). These mutants were then transiently expressed in *N. benthamiana* leaves. The results showed that the cell death-inducing ability of SRE1^67A^, SRE1^105A^, and SRE1^120A^ was significantly diminished. However, SRE1^69A^ and SRE1^87A^ were still able to cause cell death (Figure 5A). Immunoblot analysis confirmed that protein expression was successful (Figure 5A). Taken together, the Y67, R105, and H120 active sites of SRE1 are essential to causing cell necrosis. Thus, the cytotoxicity of SRE1 is contingent upon its RNase activity.

### The N-Terminal β-Hairpin Is Required for SRE1 Re-entry Into Cells to Cause Cell Death

Previous studies have demonstrated that the N-terminal of secreted RNase plays a critical role in cytotoxicity, in particular, the loop structure formed by the N-terminal β-hairpin, which has been shown to function as a ribosome binder and is presumed to be involved in cellular uptake (Garcia-Ortega et al., 2002; Lacadena et al., 2007). The SRE1^SP−Δ18−39^ mutant (a mature SRE1 reduced the 18–39 amino acid sequence) was produced in *N. benthamiana* to explore the N-terminal β-hairpin structure related to cytotoxicity. The ability of SRE1^SP−Δ18−39^ to cause cell death in *N. benthamiana* was not affected, as demonstrated by these findings (Figure 5B). In contrast, we also mutated the active sites Y67 of SRE1^SP−Δ18−39^ and SRE1^SP^ to alanine (SRE1^SP−^^Δ18−39−67*A*^ and SRE1^SP−67A^) and then expressed them in *N. benthamiana*. Eventually, we discovered that these two mutants with a mutation in the enzyme's active site lost all cytotoxicity in *N. benthamiana* (Figure 5B). Immunoblot analysis confirmed that protein expression was successful (Figure 5B). These findings suggest that RNase activity is essential for SRE1 cytotoxicity, but the N-terminal β-hairpin is not required when SRE1 is only produced intracellularly and not released.

Although SRE1 is a secretable protein and cytotoxicity occurs inside the cell, we hypothesized that the *S. turcica* secretes SRE1 into the extracellular space of the plant and then somehow enters the cell to exert its toxicity. To demonstrate whether the N-terminal β-hairpin of SRE1 is associated with re-entry into plant cells, the signal peptides of the three proteins mentioned above were retained and obtained three new mutants, SRE1^Δ18−39^, SRE1^67A^, and SRE1^Δ18−39−67*A*^ for expression in *N. benthamiana*. As we expected, SRE1^67A^ and SRE1^Δ18−39−67*A*^ lost the ability to cause cell death due to mutations in the enzyme's active site. Surprisingly, SRE1^Δ18−39^ lost its cytotoxicity despite having an intact RNase structure. We found that the SRE1^Δ18−39^ mutant's activity was similar to that of the SRE1^67A^ catalytic mutant, which had almost completely lost the ability to cause cell death (Figure 5B). The success of protein expression was validated by immunoblot analysis (Figure 5B). These findings suggest that the N-terminal 22 amino acids of mature SRE1 are only important for plant cell toxicity when the protein is first directed to the apoplastic region. The most likely explanation for this effect is that the N-terminal β-hairpin structure may contribute to SRE1 re-entry into host cells to induce toxic function.

### SRE1 Exerts Cytotoxicity by Degrading Plant RNA

**Figure 6:**
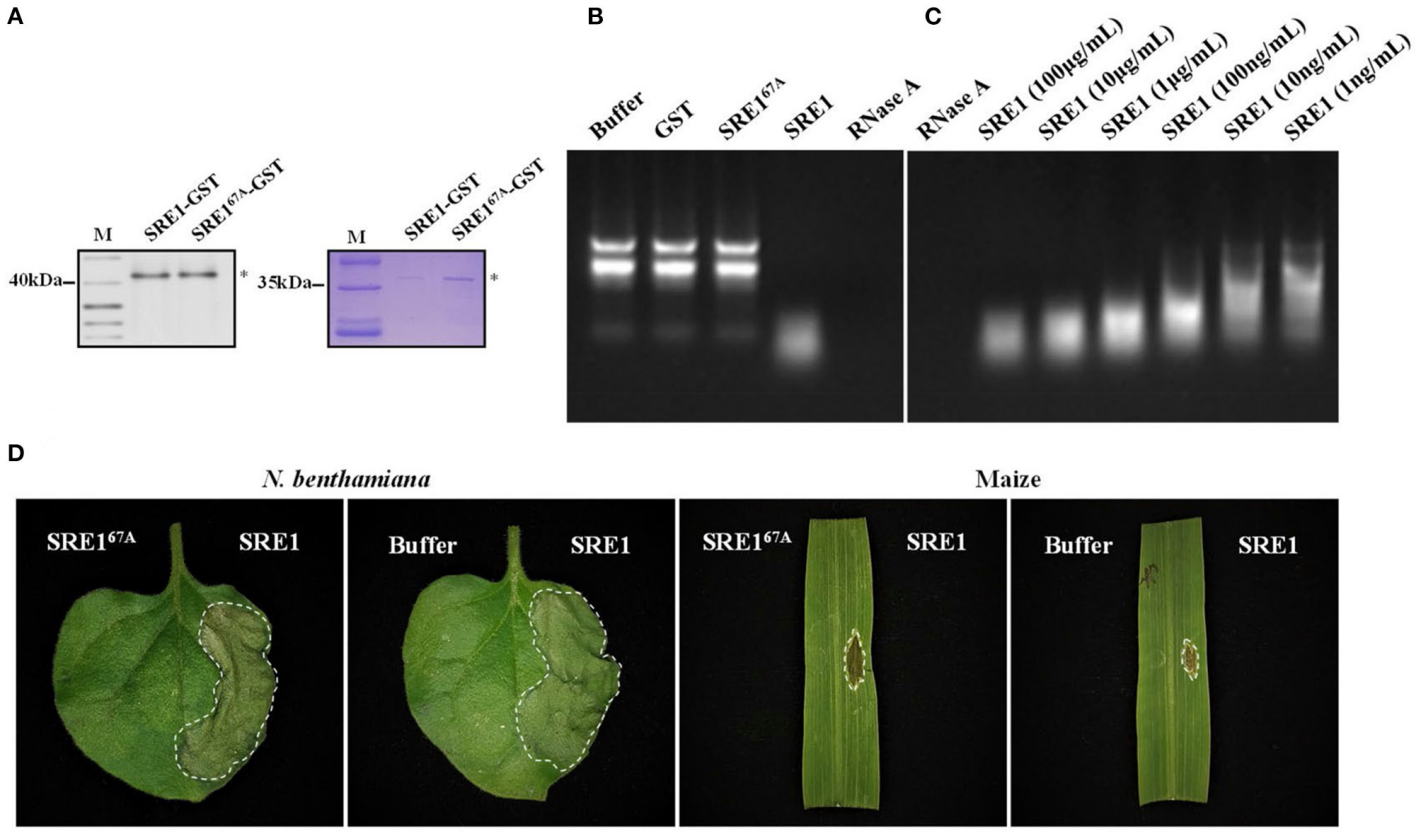
SRE1 induces cell necrosis through the degradation of total plant RNA. **(A)** Coomassie brilliant blue detection and immunoblot analysis of SRE1-GST and SRE1^67A^-GST. Asterisks represent the location of the target proteins. **(B)** Maize total RNA was used to determine the RNase activity of SRE1 and SRE1^67A^ recombinant proteins. Buffer and GST were negative controls, and RNase A was used as a positive control. The final concentrations of GST, RNase A, SRE1, and SRE1^67A^ were 100 μg/mL. **(C)** Effects of different concentrations of SRE1 on the degradation of maize total RNA. **(D)** *N. benthamiana* and maize leaves were treated with recombinant proteins SRE1 and SRE1^67A^ at a concentration of 0.4 mg/mL. The buffer solution was used as a negative control.

The SRE1 gene encodes an RNase according to protein function prediction. Recombinant protein SRE1 and mutant SRE1^67A^ were developed in the *E. coli* BL21 (DE3) strain to evaluate if SRE1 possesses RNase activity. Since the protein has a lethal effect on *E. coli*, the expression could not be induced for an extended time and increasing the fermentation culture size could obtain a small amount of protein in the supernatant. We detected the presence of SRE1 and SRE1^67A^ fused with Glutathione-S-Transferase (GST) tags by Coomassie Brilliant Blue Staining and immunoblot analysis (Figure 6A). Subsequently, the RNase activity was evaluated by incubating 1 μg of maize total RNA with recombinant proteins SRE1 and SRE1^67A^ in a 20 μl system at 25°C for 20 min. In the reaction system, the final concentration of recombinant protein was 100 μg/mL. Solution buffer and GST were negative controls, and RNase A was used as a positive control. The results demonstrated that, like the positive control RNase A, SRE1 completely obliterated total maize RNA (18s, 28s, and 5.8s rRNA). Nevertheless, the RNA electrophoresis bands remained intact after incubation of SRE^67A^ with total maize RNA, which was consistent with the negative control. Therefore, the RNase active site mutant SRE1^67A^ lost the ability to degrade plant RNA compared to SRE1 (Figure 6B). RNase activity assays were also performed using recombinant SRE1 protein at concentrations ranging from 100 μg/mL to 1 ng/mL. We found that the recombinant SRE1 degraded the RNA even when used at 1 ng/mL (Figure 6C). By infiltrating protein solutions into *N. benthamiana* and maize, we also investigated the capacity of purified SRE1 and SRE1^67A^ synthesized in *E. coli* to trigger cell death, with solution buffer as a control. The pure recombinant protein SRE1 still could cause cell death, whereas SRE1^67A^ had entirely lost its cytotoxicity and was unable to cause cell death (Figure 6D).

### SRE1 Induces Responses in *N. benthamiana*

**Figure 7:**
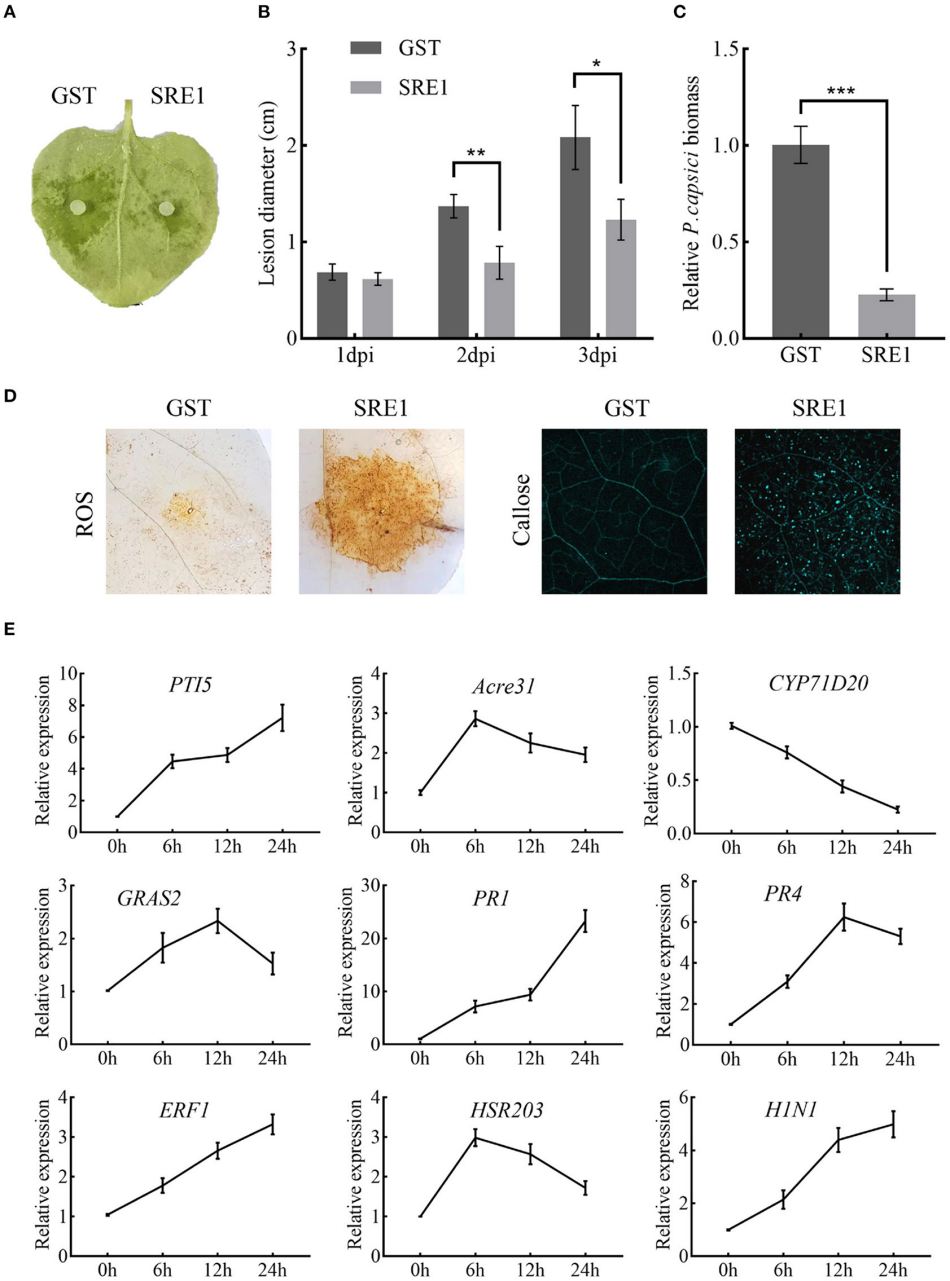
SRE1 triggers immune responses in *N. benthamiana*. **(A)** Pretreatment with 1 μg/mL SRE1 significantly increased resistance against the oomycetes pathogen *P. capsica*. The photograph was taken 2 days post-inoculation (dpi). **(B)** The lesion diameter was evaluated at 1, 2, and 3 dpi. The bars are the average of three independent experiments, and the error bars indicate standard deviations (* *P* < 0.05, ** *P* < 0.01, Student's *t*-test). **(C)** Infected leaves were collected 48 h post-inoculation, and total DNA was extracted for qPCR analysis. *NbEF-1*α was used as a reference gene, and the relative expression of *PcActin* was calculated as the relative pathogen biomass. The bars are the average of three independent experiments, and the error bars represent the standard deviations (*** *P* < 0.001, Student's *t*-test). **(D)** ROS and callose production in *N. benthamiana* leaves treated with 1 μg/ml SRE1. **(E)** Relative expression of the PTI-associated marker genes (*PTI5, Acre31, CYP71D20*, and *GRAS2*), pathogenesis-related genes (*PR1, PR4*, and *ERF1*), and HR genes (*HSR203* and *H1N1*). Relative expression was quantified by qRT-PCR using *NbEF-1*α as a reference gene. Bars indicate mean fold changes (±*SD*).

The sensitivity of SRE1-treated *N. benthamiana* to pathogens was tested to examine if SRE1 modifies plant immunity. Half of the *N. benthamiana* leaves were treated with SRE1 and the other half with GST as a control 2 h before inoculation with *P. capsici* (Figure 7A). The average lesion diameter caused by *P. capsici* on the leaves of *N. benthamiana* treated with SRE1 was significantly smaller than GST (Figure 7B). Similarly, the DNA relative biomass of *P. capsici* treated with SRE1 was also significantly lower than of the control tissue (Figure 7C). Furthermore, compared to the control, transient expression of SRE1 in plant leaves resulted in smaller lesion size and reduced DNA biomass in *P. capsici* and *B. cinerea* (Supplementary Figure 4). These findings show that treating plants with SRE1 reduces their susceptibility to fungal and oomycete infections.

To identify what causes SRE1 to reduce the susceptibility of plants to pathogens. ROS accumulation, callose deposition, and expression of defense-related genes were examined in *N. benthamiana* treated with SRE1 and GST. After 2 h of treatment of plant leaves with the recombinant protein, SRE1 treatment exhibited strong ROS accumulation and callose deposition compared to the GST control (Figure 7D). This behavior could be one of the key reasons for SRE1's ability to improve plant resistance. In addition, leaves of *N. benthamiana* that had been treated with SRE1 for 0, 6, 12, and 24 h were taken for qPCR analysis. The expression levels of the four PTI marker genes were determined (*PTI5, Acre31, CYP71D20*, and *GRAS2*) (Heese et al., 2007; Kiba et al., 2018; Wang et al., 2020), three defense-related genes in the hormone signaling pathway (*PR1, PR4*, and *ERF1*) (Dean et al., 2005; Asai and Yoshioka, 2010; Pieterse et al., 2012), and two hypersensitive response (HR) marker genes (*HSR203* and *HIN1*) (Pontier et al., 1998; Gopalan et al., 2010). qRT-PCR showed that *PR1, PR4*, and *ERF1* expression was significantly induced by SRE1. *PTI5* and *HIN1* expressions were also significantly upregulated in the 6–24 h interval. However, *Acre31, HSR203*, and *GRAS2* expression were only weakly induced by SRE1. Interestingly, *CYP71D20*, a key PTI marker gene, was not induced (Figure 7E). These results suggest that SRE1 can indeed be recognized by *N. benthamiana* and activate plant immunity, thereby enhancing plant disease resistance different from the typical PTI response.

### SRE1 Is Required for *S. turcica* Virulence in Maize

**Figure 8:**
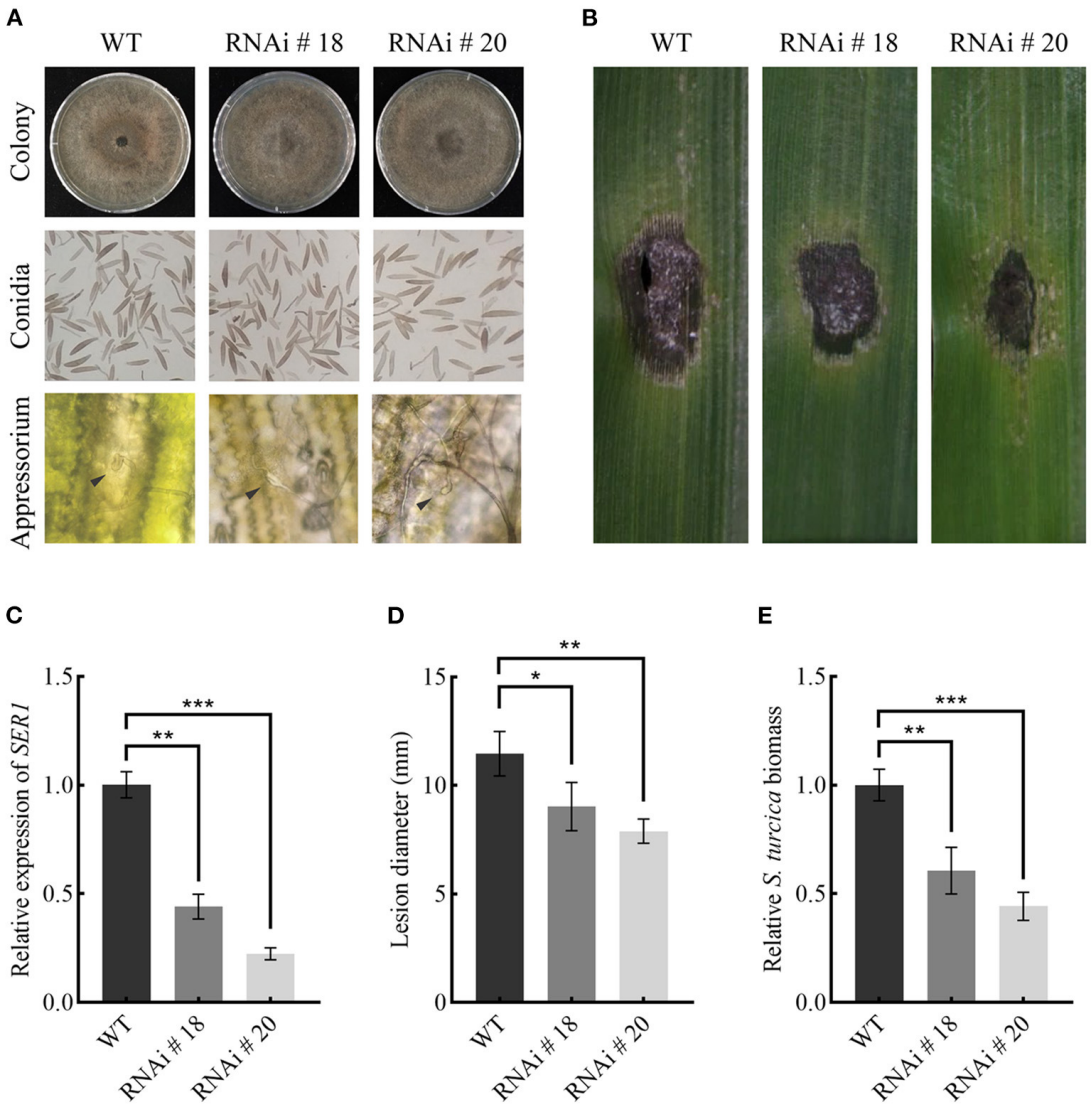
SRE1 contributes to the virulence of *S. turcica*. **(A)** Comparison of colony, conidia, and appressorium morphology between wild-type TL-5 and silent transformants RNAi#18 and RNAi#20. The black arrow in the picture points to the newly-formed appressorium. **(B)** Pathogenicity assays to investigate the role of *SRE1* gene silencing transformants RNAi#18 and RNAi#20 in the virulence of maize B73. The photograph was taken at 5 dpi. **(C)** The qPCR results showed the relative transcript levels of *SRE1* in *SRE1*-silenced transformants. Average lesion diameters **(D)** and relative *S. turcica* biomass **(E)** were calculated from three independent experiments and the error bars indicate standard deviations (*P < 0.05, **P < 0.01, ***P < 0.001, Student's *t*-test).

Transcription of *SRE1* was knocked down by using RNA interference (RNAi) to assess its role in maize infection. The target segment of *SRE1* was introduced into the pSilent-Dual1 system (Nguyen et al., 2008) to construct the silencing vector pSD1-*SRE1* (Supplementary Figure 5). The plasmid was then transformed into the protoplasts of the wild-type strains via the polyethylene glycol (PEG)-mediated method (Liu et al., 2018; Zeng et al., 2020). Finally, two stable transformants, RNAi#18 and RNAi#20 were obtained (Figure 8A). PCR tested the antibiotic-resistant transformants for the presence of the antibiotic marker gene (Supplementary Figure 6). The qPCR analysis revealed that the *SRE1* gene silencing efficiency of RNAi#18 and RNAi#20 relative to the wild-type strains was 56 and 78%, respectively (Figure 8C). The colony and conidia morphology of the silenced transformants were not significantly different from those of the wild-type strains (Figure 8A). The formation of the appressorium was not affected compared to the wild-type strains (Figure 8A). However, the knockdown mutants caused milder symptoms in maize leaves than the wild-type strains 5 days after inoculation (Figure 8B). The average lesion diameter was further counted; the results showed that the lesion diameter of maize leaves inoculated with *SRE1* knockdown mutants was significantly smaller than that of wild-type strains (Figure 8D). We evaluated the ratio of *S. turcica* DNA to maize DNA using qPCR to determine the biomass of *S. turcica* in infected maize leaves; the biomass of the *SRE1* knockdown mutants in maize leaves was considerably lower than that of the wild-type strains (Figure 8E). These findings suggest that SRE1 plays an important role in *S. turcica* pathogenicity during maize infection.

## Discussion

*S. turcica* can cause maize leaf blight, resulting in significant yield losses. Previous research analyzed the genomic properties of 18 Dothideomycete members, examining their genomic architecture, evolution, and varied pathogenic tactics. *S. turcica* was included in these investigations which is estimated to possess 214 small secreted proteins (Condon et al., 2013), but only a few are known to be involved in the infection process. Studies have shown that many effectors associated with pathogenicity were identified by analyzing the secretome of plant pathogens (Anderson et al., 2017; Zhang et al., 2017). *S. turcica's* secretory proteome was studied, and 90 effector proteins were discovered. Most identified proteins belong to enzymes with different functions, including glycosyl hydrolases, oxidoreductases, peptidases, carbohydrate esterases, proteases, isomerases, and RNases (Supplementary Table 1). SRE1, one of the effectors secreted by *S. turcica* that piqued our interest, was predicted to serve as an extracellular RNase, and we discovered that *SRE1* was highly increased in transcriptome-sequencing data from maize infected with *S. turcica* isolate TL-5 (unpublished content). *SRE1* encodes a fungus-secreted RNase of 135 amino acids, which is predicted to contain a fungal type RNase domain (Supplementary Figure 1). SRE1 triggers plant immune responses (Figure 7), leading to cell death in *N. benthamiana* and maize (Figure 6). Similarly, SRE1 homologs in other phytopathogenic fungi can also cause cell death in *N. benthamiana* leaves (Figure 3). As a result of our findings, SRE1, a ubiquitous cell death-inducing effector with RNase activity, is activated during *S. turcica* infection and leads to pathogenesis (Figure 8).

The cell death caused by effectors has been previously reported in many pathogens. Studies have shown that secreted small cysteine-rich proteins (SCPs) modulate host immunity in pathogen-plant interactions, and some VdSCPs of *V. dahliae* can induce cell death in *N. benthamiana*, play a key role in *V. dahliae*-plant interactions through intrinsic virulence functions, and suppress immunity after infection (Wang et al., 2020). VmE02 is a cell death inducer isolated from *V. mali* and later identified as a novel transboundary PAMP with a wide distribution in oomycetes and fungi (Nie et al., 2019). A recent study has shown that StACE1 acts as an appressorium-coupled effector that can cause cell necrosis in *N. benthamiana* (Meng et al., 2022). There is, however, limited research on *S. turcica* effectors or PAMPs, which can trigger cell death and control host immunity. We used agroinfiltration on *N. benthamiana* leaves and discovered that SRE1 caused cell death in the plant. The HR elicited by the elicitor INF1 was similar to this cell necrosis phenomenon (Figure 3A). Although we speculate that the cytotoxicity of SRE1 is likely due to the direct cleavage of *N. benthamiana* rRNA by the RNase activity of SRE1, we cannot exclude the host plant actively recognizing the possibility of the necrosis induced by SRE1. Currently, there is no relevant report on the recognition receptor of RNase in plants, so we need to further verify it through experiments. A recent report indicates that this mechanism of RNase-induced cell death is similar to the well-known Nep1-like proteins (NLPs) in many phytopathogens, which act as both cytotoxic and elicitors of plant innate immune responses (Qutob et al., 2006; Van den Ackerveken, 2017; Steentjes et al., 2022; Yin et al., 2022).

The yeast signal capture assay (Jacobs et al., 1997) demonstrated that the signal peptide of SRE1 did have a secretory function (Figure 2A). Initially, we hypothesized that SRE1 could be secreted into the apoplast space of the host plant to perform the corresponding function. However, when the signal peptide of SRE1 was truncated, we found that even if SRE1 was not secreted, it could still lead to plant cell death (Figure 3A). As a result, we concluded that SRE1's cytotoxicity could be exerted in the intracellular space. Effector-induced cell death seemed to be the outcome of cell surface immune receptors actively recognizing effectors in the apoplast. RXEG1, for example, binds to XEG1 in the apoplast via the LRR domain and forms a complex with the LRR receptor-like kinases BAK1 and SOBIR1 to transduce XEG1-induced defense signals (Wang et al., 2018). In *V. dahliae*, VdEG1 and VdEG3 trigger PTI in different ways. BAK1 is required for VdEG1 and VdEG3-triggered immunity, while SOBIR1 is specifically required for VdEG1-triggered immunity in *N. benthamiana* (Gui et al., 2017). In the process of screening potential recognition receptors of *F. oxysporum* cell wall extract (FoCWE), two receptor-like proteins (GhRLP20 and GhRLP31) in cotton were found to be required for the recognition of FoCWE and *Fusarium* resistance (Babilonia et al., 2021). SRE1-induced plant cell death was independent of BAK1 and SOBIR1 in this study (Figure 4). As a result, these plant immunological receptors in the apoplast do not recognize SRE1. SRE1 is unlikely to be an apoplastic effector, based on these observations. SRE1 is found in a wide range of phytopathogenic fungi with various lifestyles, according to phylogenetic analyses (Figure 2B). Homologs of SRE1 also caused plant cell necrosis in *N. benthamiana*, but Fg12 of *F. graminearum* (Yang et al., 2021), FoSRE1 of *F. oxysporum*, and CgSRE1 of *C. graminicola* were signal peptide-dependent (Figure 3A). However, the mechanism for this difference in the signal peptide dependence remains to be elucidated further. We hypothesize that plants may have evolved new ways to recognize specific RNases during co-evolution with pathogens, activating immune responses and inducing cell death, such as the presence of unique recognition receptors on the cell membrane. Perhaps these signal peptide-independent RNases evolved novel activities other than intracellular toxicity.

Studies have revealed that the RNase enters the cell and cleaves the SRL loop on the large ribosomal subunit, which resembles a twisted hairpin (Makarov and Ilinskaya, 2003). This cleavage results in the inhibition of protein biosynthesis, followed by cell death by apoptosis (Olmo et al., 2001). Each active site was mutated to study whether SRE1-induced cell death is dependent on enzymatic activity. The findings revealed that mutations at Y67, R105, or H120 impact the protein's cytotoxicity (Figure 5A). The cytotoxicity of Fg12 in *F. graminearum* and UV_1423 in *U. virens* is likewise affected by the RNase active site (Fang et al., 2016; Yang et al., 2021).

Meanwhile, SRE1 and mutant SRE1^67A^ were obtained by prokaryotic expression. SRE1 has non-specific RNase activity against maize total RNA, similar to that of RNase A, according to our findings. The SRE1^67A^ mutant, on the other hand, was unable to degrade maize total RNA (Figure 6B). We also tested the ability of purified SRE1 and SRE1^67A^ mutant to induce cell death by infiltrating protein solution into *N. benthamiana* and maize leaves. As expected, the SRE1^67A^ mutant showed impaired cytotoxicity to *N. benthamiana* and maize (Figure 6D). Therefore, we speculated that SRE1 cytotoxicity depended on its degradation of plant intracellular RNA, which may include the rRNA in the ribosomes. In addition, another study showed that *Blumeria graminis* could also secrete an RNase-like effector protein CSEP0064/BEC1054, which lacks the catalytic triad present in RNase F1. As a result, it functions as a pseudoenzyme, protecting the host rRNA from destruction and facilitating fungal nutrition acquisition from living cells (Pennington et al., 2019).

According to research, the N-terminal β-hairpin structure of the ribosomal toxin protein -sarcin possesses many positive charges associated with the cellular lipid membrane interaction (Garcia-Ortega et al., 2002). Although hirsutellin A and restrictocin have a shorter N-terminal β-hairpin, this flaw appears to be compensated for by the elongation of loop 5, which also has a greater positive charge (Martínez del Pozo et al., 1988; Olombrada et al., 2017). In *Z. tritici*, the RNase Zt6 relies on its N-terminal loop to enter the cell (Kettles et al., 2018). In this study, SRE1 lost its ability to cause cell necrosis when it contained a signal peptide and lacked the β-hairpin structure formed by amino acids 18-39 at the N-terminus (Figure 5B). We assume that the absence of the β-hairpin structure inhibits SRE1 from entering the cytoplasm and performing its toxic activity. SRE1 differs from the previously reported Fg12 in that Fg12 is presumed to degrade RNA in the extracellular space of plants, thereby promoting fungal infection (Yang et al., 2021) since previous studies have shown that pathogens can absorb small RNAs in the apoplast to inhibit the expression of the virulence genes (Wang et al., 2016; Cai et al., 2018).

Pathogens developed the ability to deliver effector proteins to interfere with PTI through the co-evolution of host-microbe interactions, allowing pathogens to invade and cause disease in their particular host plants (Wang et al., 2022). For instance, the conserved effector protein NIS1 in fungi specifically targets and reduces the kinase activity of BAK1 and BIK1, inhibits cell necrosis and reactive oxygen burst caused by PAMPs, and thus positively regulates the pathogenicity of fungi (Irieda et al., 2019). In this study, SRE1 recombinant protein improved the resistance of *N. benthamiana* to *P. capsici* and *B. cinerea* (Figures 7A–C and Supplementary Figure 4), especially by promoting ROS production and the accumulation of callose (Figure 7D). In addition, SRE1 can activate and induce the expression of defense-related genes (*PR1, PR4*, and *ERF1*) and genes related to hypersensitive necrosis response. However, SRE1 only weakly activates PTI-related marker genes compared to other typical PAMPs (Figure 7E). As a result, we infer that SRE1-induced cell death differs from the conventional PTI immune response and that the plant's immune response may be initiated during RNase degradation of intracellular RNA.

We evaluated the virulence of SRE1 during infection with *S. turcica*. After gene silencing of *SRE1*, we found that the morphology and infection structure of *S. turcica* did not change (Figure 8A), indicating that SRE1 may not be directly involved in regulating the growth and development of *S. turcica*. During the early stages of infection, though, silencing SRE1 lowered the virulence of *S. turcica in* maize leaves (Figures 8B–E). *S. turcica* SRE1 may be a potential virulence factor that causes host cell death. Therefore, this strategy helps *S. turcica* rapidly transition to the necrotrophic stage and colonize the host. Similarly, the RNase Fg12 from *F. graminearum* also displays significant pathogenicity in soybean hypocotyls (Yang et al., 2021). In comparison, the lack of Zt6 did not appear to decrease *Z. tritici* pathogenicity, and given that Zt6 was the only RNase found in the secretome of *Z. tritici*, functional redundancy is unlikely to be the cause of the lack of virulence loss phenotype. However, based on the results, Zt6 may have an important function in antimicrobial competition and niche protection rather than cytotoxicity on the host (Kettles et al., 2018). In summary, SRE1 contributes to the pathogen's virulence as an RNase effector secreted by *S. turcica*, which can cause plant cell death relying on its enzymatic and activate plant immunity to enhance plant resistance.

## Data Availability Statement

The datasets presented in this study can be found in online repositories. The names of the repository/repositories and accession number(s) can be found in the article/Supplementary Material.

## Author Contributions

SH designed and wrote the manuscript. All authors contributed to the article and approved the submitted version.

## Funding

This work was supported by the National Key Technology Research and Development Program of China under Grant Nos. 2017YFD0300704, 2016YFD0300704, and 2018YFD0300307, and the Natural Science Funds of Hebei Province (C2019402430).

## Conflict of Interest

The authors declare that the research was conducted in the absence of any commercial or financial relationships that could be construed as a potential conflict of interest.

## Publisher's Note

All claims expressed in this article are solely those of the authors and do not necessarily represent those of their affiliated organizations, or those of the publisher, the editors and the reviewers. Any product that may be evaluated in this article, or claim that may be made by its manufacturer, is not guaranteed or endorsed by the publisher.
