# Supplementary material for: The Secreted Ribonuclease SRE1 Contributes to Setosphaeria turcica Virulence and Activates Plant Immunity
Source: Front Microbiol. 2022 Jul 8;13:941991. doi: 10.3389/fmicb.2022.941991 (PMC9304870; doi:10.3389/fmicb.2022.941991)
Supplement: Supplementary file 2 [file Data_Sheet_1.PDF]

The supplementary Figures

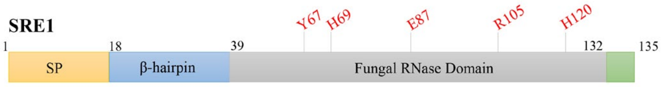

Supplementary Figure 1. The schematic illustration of SRE1

The yellow, blue, and gray blocks represent the Signal Peptides (SP), N-terminal β-hairpin, and Fungal RNase Domain, respectively. The red letters represent enzyme active sites (67Y, 69H, 87E, 105R, and 120H).

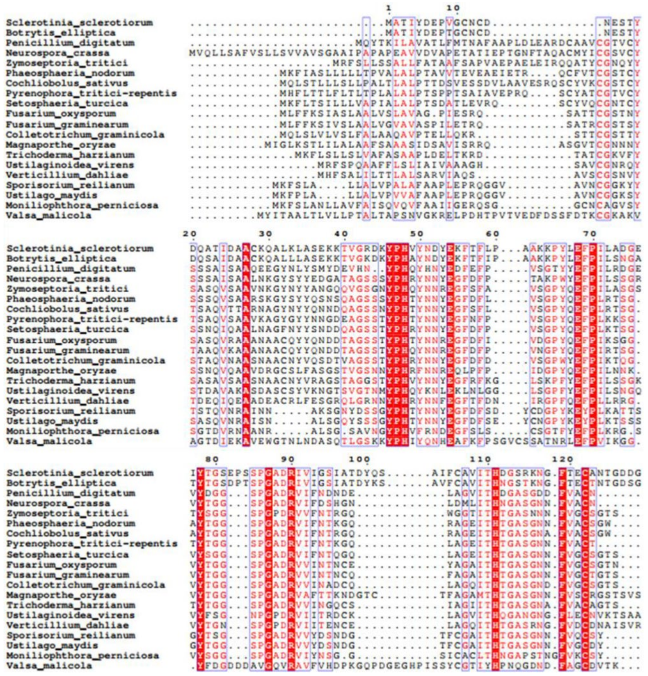

Supplementary Figure 2. Bioinformatics analysis of the RNase family in fungi.

Sequence alignment of *Setosphaeria turcica* SRE1 with known RNases from other fungi. RNase sequences in different fungi were analyzed by ESPript 3.0. The results show that the catalytic domains of RNase in different fungi are very conserved, but the N-terminal sequence has a high degree of variation.

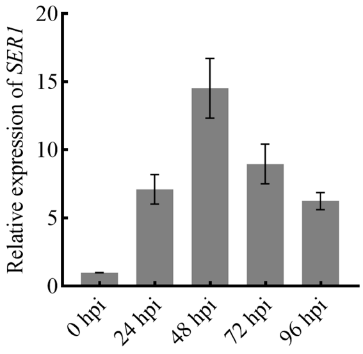

Supplementary Figure 3. *SRE1* expression profile of *S. turcica* during the infection

The relative transcript levels of *SRE1* at different infection stages of *S. turcica* were assessed by qPCR. Maize leaves were sampled 24, 48, 72, and 96 h after being inoculated with wild-type TL-5. *StTublin* was used as an internal reference gene and the relative expression level of mycelium (0h) was considered as 1. The bars are the average of three independent experiments and the error bars indicate the standard deviation.

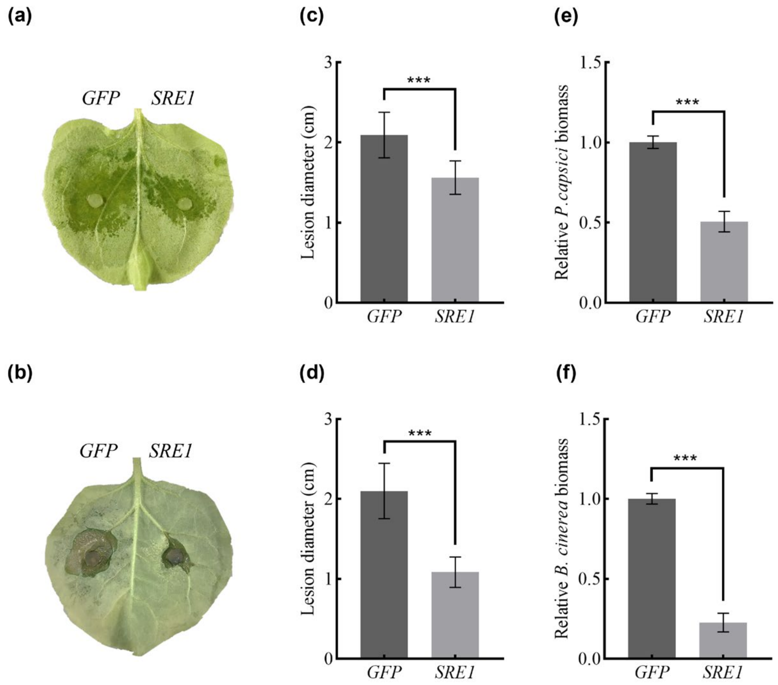

Supplementary Figure 4. Transient expression of SRE1 significantly increased *N. benthamiana* resistance against oomycete and fungal pathogens

(a, b) Representative *N. benthamiana* leaves show lesions caused by *Phytophthora capsici* and *Botrytis cinerea*, respectively. *A. tumefaciens* cultures harboring GFP and SRE1 were injected into the inoculation sites on both sides of *N. benthamiana* leaves 24 hours in advance. The photographs were taken at 2 dpi. Average lesion diameters (c, d) and relative pathogen biomass (e, f) were calculated from three independent experiments. The error bars indicate standard deviation (*** $P < 0.001$, Student's t-test).

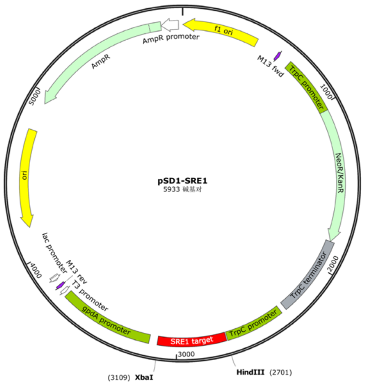

SRE1 RNAi sequence

AAGTTCCTCACTAGCATCCTGCTCTTGGTCGCGCCCATTGCCCTTG
CCCTCCCTACTTCCGATGCCACCCTGGAGGTGAGGCAGAGCTGCT
ACGTGCAATGCGGTAACACCTGCTACTCGTCGAACCAAATCCAAG
CTGCTCTCAACGCCGGATTCAACTACTACAGCAACGACGACCAGG
CTGGCAGCTCGACGTACCCTCACAGGTACAACAACTACGAGGGAT
TCGACTTCCCCGTCTCCGGCCCGTACCAGGAGTTTCCGCTCAAGA
CTTCGGGTGTCTACAGTGGCGGATCTCCTGGTGCCGACCGTGTCA
TCTTCAACACTCAGGGCCAGTTGGCTGGAGAGATTACACACACTG
GTGCTAGTGGCAACAACTTTGTTGGTTGCTCGGGAACTTCG

Supplementary Figure 5. Map of pSilent-Dual1-SRE1 constructs

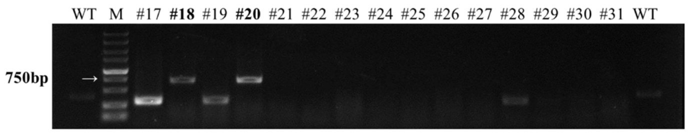

Supplementary Figure 6. Identification of gene-silencing transformants by PCR
